# Stress index (glucose-to-potassium ratio) as a predictor of outcome in traumatic brain injury: systematic review and meta-analysis

**DOI:** 10.3389/fneur.2026.1848006

**Published:** 2026-10-01

**Authors:** Mesum Abbas, Muhammad Moin-Ud-Din Arshad, Ali Sher, Rimsha Maqsood, Muhammad Bilal Akram, Areena Fatima, Sadat Ali, Waqar Ali Shah, Muhammad Anees, Abdullah Faramarz, Abdur Rehman, Muhammad Abdullah Masood, Amit Kumar Thakur

**Affiliations:** 1 Quaid-e-Azam Medical College, Bahawalpur, Pakistan; 2 Sukrajaj Hospital of Tropical and Infectious Disease, Kathmandu, Nepal

**Keywords:** traumatic brain injury, glucose-to-potassium ratio, stress index, prognosis, mortality, biomarker, systematic review, meta-analysis

## Abstract

**Background:**

Traumatic brain injury (TBI) is a major cause of death and disability. The glucose-to-potassium ratio (GPR), or stress index (SI), reflects stress hyperglycemia and hypokalemia and may improve early prognostication beyond clinical and radiologic scores.

**Methods:**

We performed a PRISMA-guided systematic review and meta-analysis of observational cohort studies in adults (≥18 years) with CT-confirmed TBI reporting admission glucose and potassium and their association with mortality or functional/“poor outcome.” Random-effects models pooled odds ratios (ORs) for dichotomous outcomes and mean differences (MDs) for continuous GPR values; heterogeneity was assessed with I^2^. Prespecified subgroup analyses were conducted by sample size (>500 vs. < 500), with sensitivity analyses (leave-one-out; fixed vs. random) and funnel-plot inspection to explore robustness and small-study effects.

**Results:**

Three studies (4,550 patients) contributed to the primary comparison of high versus low GPR and mortality. High GPR was associated with increased odds of death (pooled OR 11.97, 95% CI 2.68–53.37; *p* = 0.001; I^2^ = 88%). Across four studies, patients with poor outcomes had higher admission GPR than those with good outcomes (pooled MD 16.82, 95% CI 12.70–20.94; *p* < 0.00001; I^2^ = 61%). In five studies evaluating GPR as a prognostic predictor, the pooled adjusted OR for adverse outcome was 2.10 (95% CI 1.51–2.93; *p* < 0.0001; I^2^ = 37%). Effects were larger in small single-center cohorts and more moderate, but significant, in the large registry-based study; sample size explained much of the between-study heterogeneity. Funnel-plot asymmetry suggested possible small-study effects/publication bias.

**Conclusion:**

Elevated admission GPR/SI is associated with increased mortality and poor outcome in adult TBI and may capture early systemic stress. As a bedside metric derived from routine laboratory tests, it is a promising adjunct for early risk stratification. However, heterogeneity, small-study effects, and observational evidence warrant cautious interpretation; large multicenter prospective studies are needed to validate standardized thresholds and incremental value over existing prognostic models.

## Introduction

Traumatic brain injury (TBI) is a leading cause of death and long-term disability worldwide, contributing substantially to years of life lost and years lived with disability in both high-income and low- and middle-income countries. Large Global Burden of Disease analyses estimate tens of millions of new TBI cases annually, with rising incidence in many regions due to road traffic injuries, falls, and interpersonal violence (1–3). Despite advances in pre-hospital care, neuromonitoring, and neurocritical care, mortality and severe disability remain high, particularly among patients with moderate-to-severe TBI (2, 4).

Current prognostic assessment is based on clinical scales such as the Glasgow Coma Scale (GCS), pupillary responses, radiological classifications (e.g., Marshall and Rotterdam CT scores), and validated prognostic models such as the Corticosteroid Randomisation After Significant Head Injury (CRASH) model, often integrated into prognostic models and guideline-based algorithms for severe TBI (2, 4, 5). However, these tools may be limited by inter-observer variability, incomplete availability at first contact, and their focus on structural or neurological variables rather than on the systemic stress response. In the emergency department, clinicians must often triage and make early decisions on intubation, neurosurgical intervention, and ICU admission within minutes, using simple tests that are globally accessible. There is therefore continuing interest in inexpensive, rapidly obtainable biochemical markers that could complement established clinical predictors (4, 5).

One of the most consistent biochemical phenomena in acute TBI is stress-induced hyperglycemia, driven by catecholamine and cortisol surges, insulin resistance, and inflammatory signaling (6). Observational data suggest that elevated admission or early glucose levels are independently associated with higher mortality, unfavorable functional outcome, and increased risk of complications after TBI, even after adjustment for injury severity (6–8). Earlier evidence also demonstrated the prognostic significance of admission hyperglycemia in neurocritical care settings. Prisco et al. (9) reported that elevated blood glucose levels were among the earliest predictors of mortality in patients with traumatic brain injury, supporting the concept that stress-induced metabolic dysregulation reflects injury severity and secondary brain injury mechanisms. These findings provide an important biological rationale for investigating composite glucose-based biomarkers such as the glucose-to-potassium ratio (GPR), which may better capture the overall physiological stress response following TBI. A recent systematic review and meta-analysis confirmed that both hyperglycemia and intensive glycemic variability are linked to worse neurological outcomes, although tight glycemic control has not consistently improved survival (10). These findings support the concept of hyperglycemia as a marker of global physiological stress rather than a purely modifiable target. While admission hyperglycemia alone is a well-established predictor of poor outcomes in TBI, it reflects only one dimension of the systemic stress response. The glucose-to-potassium ratio (GPR) integrates both metabolic and electrolyte disturbances, potentially providing a more comprehensive prognostic indicator.

Potassium homeostasis is closely intertwined with this stress response. Catecholamine-mediated stimulation of β2-adrenergic receptors and insulin secretion promotes intracellular potassium shifts, frequently resulting in hypokalemia during acute critical illness (11). In TBI, multiple cohort studies have documented frequent sodium and potassium disturbances on admission and during ICU stay, with associations between dyskalemia and intracranial hypertension, arrhythmias, and mortality (12–15). Hypokalemia and rapid potassium shifts have also been linked to increased mortality in general trauma populations (16, 17). These data suggest that the combination of stress hyperglycemia and dyskalemia might capture the severity of the neuroendocrine and systemic response to brain injury more accurately than either variable alone.

Building on this rationale, the serum glucose-to-potassium ratio (GPR), also known as the “stress index” (SI), has emerged as a composite biomarker that integrates hyperglycemia and hypokalemia into a single metric [glucose (mg/dL)/potassium (mEq/L)]. Initially evaluated in acute brain injury-related diseases such as intracerebral hemorrhage (ICH), GPR was shown to discriminate patients with poor 90-day outcomes better than glucose or potassium alone and to improve prognostic models based on hematoma volume or GCS (18). More recently, studies in elderly emergency department patients, sepsis cohorts, and large unselected trauma registries have reported that higher admission GPR or SI independently predicts short-term mortality and adverse outcomes (19–23). These findings support the generalizability of GPR/SI as a simple stress biomarker across diverse acute care populations.

In TBI specifically, several single-center cohorts and registry studies have evaluated GPR or SI measured on admission as a prognostic marker. Shibata et al. (24) first reported that a serum GPR ≥ 50 at hospital arrival was associated with four-fold higher odds of in-hospital death or vegetative state among patients with severe TBI. Marini et al. (25) subsequently demonstrated, in a Latin American cohort of moderate-to-severe TBI, that GPR > 50 was strongly associated with unfavorable outcome at 6 months and might provide additive prognostic value over CT-based scores. Huang et al. (26) analyzed patients with isolated moderate-to-severe TBI and found that higher SI (glucose/potassium ratio) independently predicted in-hospital mortality, suggesting that SI reflects systemic stress beyond conventional severity indices. Additional studies in patients undergoing emergency craniotomy, in mild-to-moderate TBI, and in isolated blunt head trauma have reported similar associations between higher admission GPR and increased mortality or poor functional outcome, and in some cases have compared GPR with established trauma scores (27–29). A commentary from neurotrauma experts has further highlighted the potential utility of GPR while calling for larger confirmatory studies and careful assessment of cut-off values (30).

Despite this emerging body of evidence, there has been no prior systematic review and meta-analysis focused specifically on the prognostic value of GPR/SI in adult TBI. The published studies differ in design, sample size, ethnic background, GPR/SI cut-offs, and outcome definitions, leaving uncertainty about the magnitude and consistency of the association across settings. Furthermore, the extent to which small-study effects, publication bias, and between-study heterogeneity influence pooled estimates remains unclear.

Accordingly, we conducted a systematic review and meta-analysis of observational studies assessing admission GPR or SI in adult patients with TBI. Our primary objective was to quantify the association between high versus low GPR/SI and mortality. Secondary objectives were to compare mean GPR/SI levels between outcome groups, evaluate adjusted prognostic performance, explore heterogeneity through subgroup analyses by sample size and ethnicity, and assess potential publication bias.

## Methodology

### Study design and registration

This systematic review and meta-analysis was conducted in accordance with the Preferred Reporting Items for Systematic Reviews and Meta-Analyses (PRISMA) guidelines (31). The study protocol was designed to evaluate the prognostic value of the Glucose-to-Potassium Ratio (GPR), also referred to as the Stress Index (SI), in patients with Traumatic Brain Injury (TBI). This review was not prospectively registered in PROSPERO. The absence of protocol registration should be considered when interpreting the findings, as it may limit methodological transparency and increase the potential risk of selective reporting.

### Search strategy

A comprehensive literature search was performed across major electronic databases (PubMed, Scopus, Web of Science, and Embase) to identify relevant observational studies. Keywords used included “Traumatic Brain Injury,” “TBI,” “Glucose-to-Potassium Ratio,” “GPR,” “Stress Index,” “Prognosis,” and “Mortality.” Reference lists of included articles were also manually screened to identify additional eligible studies. The search strategy was developed and conducted by the study investigators. No professional medical librarian participated in the design or validation of the search strategy. Boolean operators (“AND” and “OR”) were used to combine search terms appropriately, with “OR” used for synonyms and “AND” used to combine key concepts.

### Inclusion and exclusion criteria

Studies were selected based on the following criteria:

- Inclusion Criteria:

- Population: Adult patients (aged ≥18 years) diagnosed with Traumatic Brain Injury (mild, moderate, or severe) confirmed by neuroimaging (CT scan).
- Exposure: Measurement of serum glucose and potassium levels upon admission or arrival at the emergency department to calculate the Glucose-to-Potassium Ratio (GPR).
- Comparator: Comparison between High GPR vs. Low GPR groups, or survivors vs. non-survivors.
- Outcome: Assessment of mortality (30-day or in-hospital), functional outcome (Glasgow Outcome Scale [GOS] or modified Rankin Scale [mRS]), or hospitalization requirements.
- Study Design: Retrospective or prospective observational cohort studies.

- Exclusion Criteria:

- Polytrauma: Patients with severe extracranial injuries (e.g., burns, drowning, hemorrhagic shock) that could independently alter metabolic markers or mortality risk.
- Pediatric Population: Patients under the age of 18, as physiological baselines for GPR differ in children.
- Missing Data: Studies lacking sufficient data to calculate the GPR or extract outcomes (OR, Mean Difference).
- Confounding Comorbidities: Some studies excluded patients with pre-existing conditions that significantly alter glucose/potassium metabolism, such as diabetic ketoacidosis or chronic renal failure, though this varied by individual study protocol.

A PRISMA Flow diagram is given in Figure 1.

**Figure 1:**
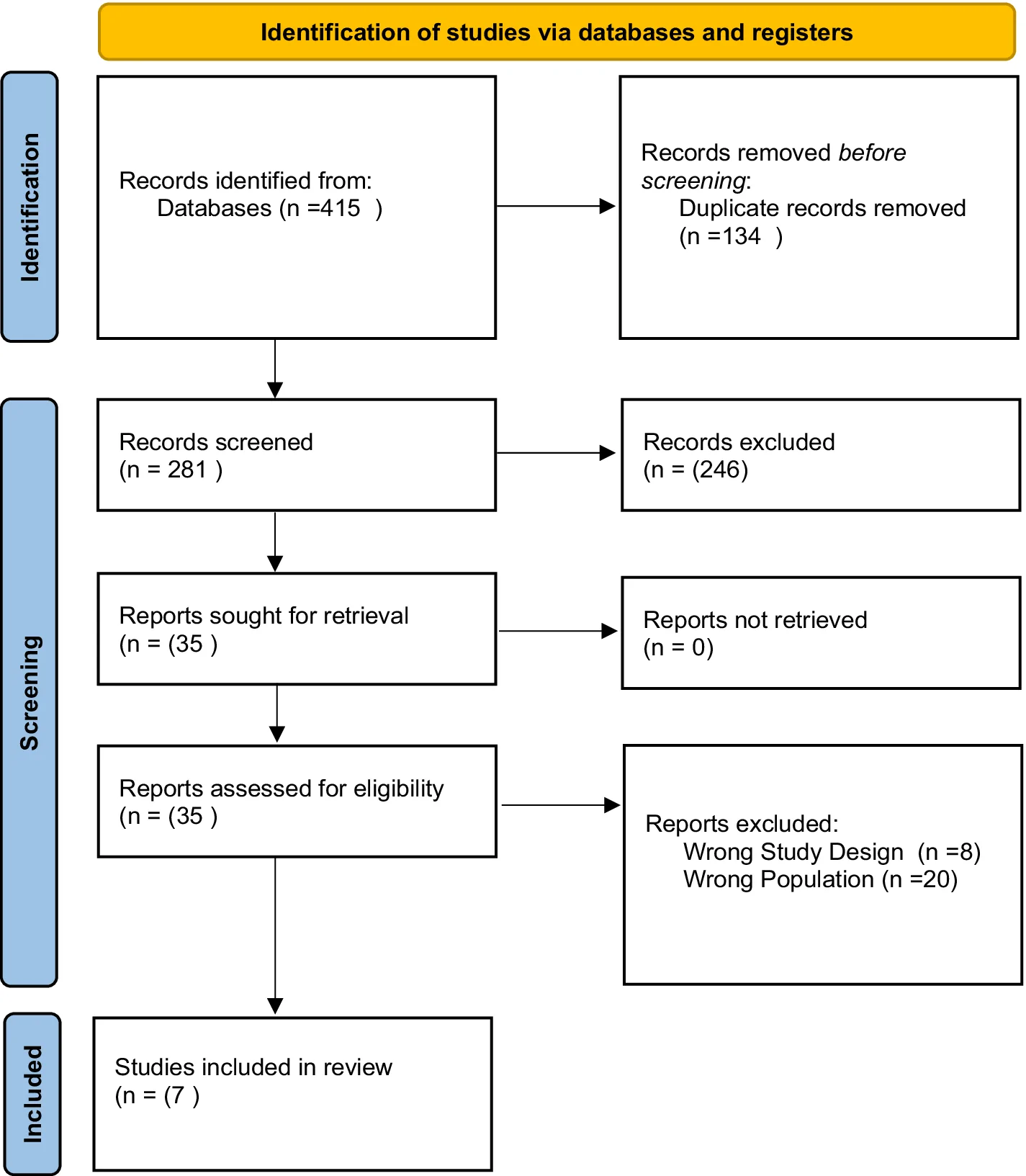
PRISMA 2020 flow diagram illustrating the literature search, screening, eligibility assessment, and final inclusion of studies for the systematic review and meta-analysis.

### Data extraction and quality assessment

Two independent reviewers extracted data using a standardized form. Extracted data included: author, year of publication, country of study, sample size, GPR cutoff values (ranging from 2.28 to 50 depending on measurement units), and outcomes. The quality of included studies was assessed using the Newcastle-Ottawa Scale (NOS) for cohort studies, evaluating selection, comparability, and outcome assessment. A Risk of bias table is given in Table 1.

**Table 1:** Risk of bias assessment of included cohort studies using the Newcastle-Ottawa Scale (NOS).

Study ID	Year	Selection (Max 4*)	Comparability (Max 2*)	Outcome (Max 3*)	Total score	Quality rating
Huang et al.	2024	****	**	***	9	High quality
Marini and Sein	2023	****	**	**	8	High quality
Shibata et al.	2021	****	**	***	9	High quality
Unal and Dogan	2025	****	*	***	8	High quality
Wang et al.	2025	****	**	***	9	High quality
Zhou et al.	2023	***	*	***	7	Moderate quality
Zhou et al.	2020	****	**	***	9	High quality

### Statistical analysis

All statistical analyses were performed using Review Manager (RevMan) version 5.4.

- Effect Measures:

- For dichotomous outcomes (e.g., Mortality in High vs. Low GPR), the pooled Odds Ratio (OR) with 95% Confidence Intervals (CI) was calculated using the Mantel–Haenszel method.
- For continuous outcomes (e.g., GPR levels in Non-Survivors vs. Survivors), the Mean Difference (MD) with 95% CI was calculated using the Inverse Variance method.

- Heterogeneity:

- Heterogeneity was assessed using the *chi^2^* test and the *I^2^* statistic*. I^2^* values of 25, 50, and 75% were interpreted as low, moderate, and high heterogeneity, respectively.
- Due to significant heterogeneity observed in the primary mortality analysis (*I^2^* = 88%), a Random-Effects Model was utilized for all meta-analyses to provide a more conservative estimate.

- Subgroup Analysis:

To explore sources of heterogeneity, subgroup analyses were conducted based on:

- Sample Size: Studies were stratified into “Large Sample Size” (N > 500) and “Small Sample Size” (N < 500) to assess small-study effects.
- Ethnicity/Region: Studies were stratified into “Asian” and “Non-Asian” populations to account for potential genetic or environmental variances in metabolic baselines.

- Sensitivity Analysis:

Sensitivity analyses were performed to test the robustness of the results. This included a “Leave-One-Out” analysis, specifically excluding the largest study (26) to observe its impact on heterogeneity (*I^2^*) and pooled effect size. Model validation was also performed by comparing Random-Effects vs. Fixed-Effects models.

- Publication Bias:

Publication bias was assessed visually using funnel plots for outcomes with sufficient included studies. Asymmetry was evaluated to detect potential small-study effects or missing negative results. Formal statistical tests for funnel plot asymmetry (e.g., Egger’s regression test) were not performed due to the limited number of included studies (k < 10), where such tests are considered unreliable and underpowered.

## Results

### Association between GPR and mortality (high vs. low GPR groups)

In the primary meta-analysis comparing patients with High GPR versus Low GPR, a total of 4,550 patients were included across three studies (25–27). The pooled analysis using a random-effects model demonstrated that patients in the High GPR group had significantly higher odds of mortality compared to the Low GPR group, with a pooled Odds Ratio (OR) of 11.97 (95% CI: 2.68, 53.37; *Z* = 3.25, *P* = 0.001).

Significant heterogeneity was observed among the studies (*I^2^* = 88, *chi^2^* = 17.11, df = 2*, p* = 0.0002). The between-study variance (*τ^2^*) was calculated to be 1.46. The individual study effect sizes ranged from an OR of 4.07 (26) to an OR of 26.73 (27) (Figure 2).

**Figure 2:**
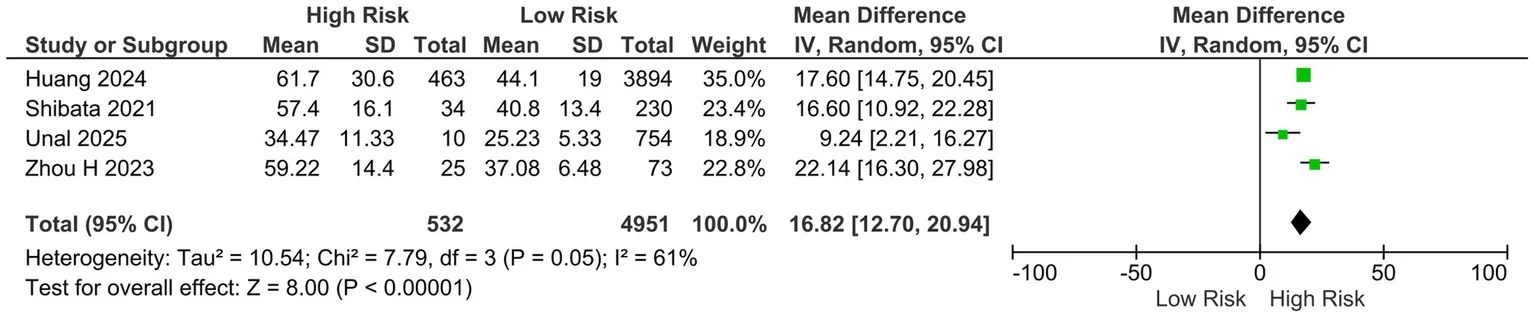
Association between high vs. low glucose-to-potassium ratio (GPR) and mortality. The analysis utilized a random-effects model due to high heterogeneity (I^2^ = 88%).

### GPR levels in high-risk vs. low-risk patients

We analyzed the mean difference (MD) in GPR levels between high-risk (poor outcome) and low-risk (good outcome) patients across four studies (24, 26, 27, 29). The pooled Mean Difference was 16.82 (95% CI: 12.70, 20.94), indicating significantly higher GPR levels in the high-risk group (*Z* = 8.00, *p* < 0.00001).

Moderate heterogeneity was present (*I^2^* = 61%, *chi^2^* = 7.79, df = 3, *p* = 0.05). The tau^2^ for this analysis was 10.54. Notably, the study by Unal (29)reported a lower mean difference (MD: 9.24) compared to Zhou (27) (MD: 22.14) (Figure 3).

**Figure 3:**
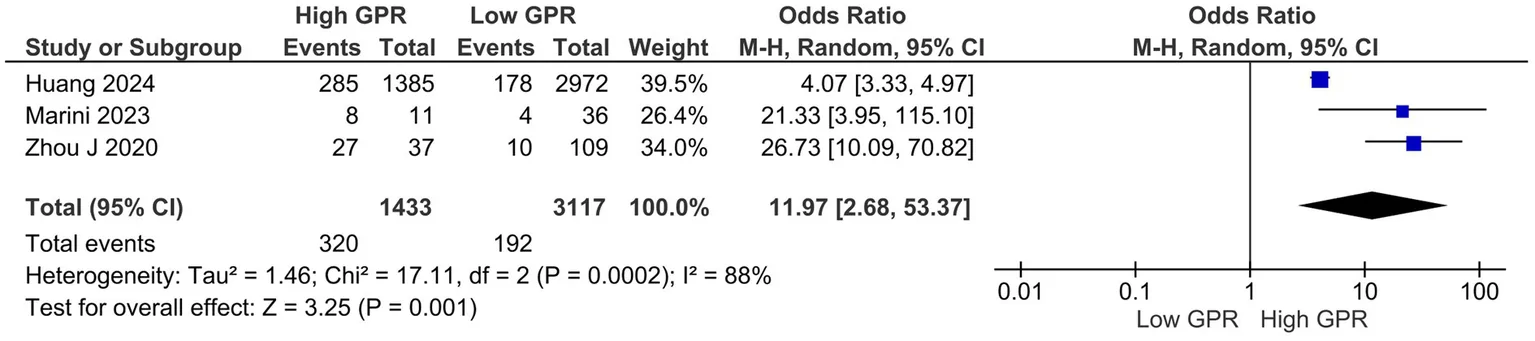
Mean difference (MD) in GPR levels between high-risk (poor outcome) and low-risk (good outcome) patients.

### Prognostic value of GPR (predictor analysis)

When analyzing GPR as a prognostic predictor across five studies (24–28), the overall pooled OR was 2.10 (95% CI: 1.51, 2.93; *Z* = 4.40, *p* < 0.0001). This analysis showed lower heterogeneity (*I^2^* = 37%, *chi^2^* = 6.38, df = 4, *p* = 0.17) compared to the high/low group comparison (Figure 4).

**Figure 4:**
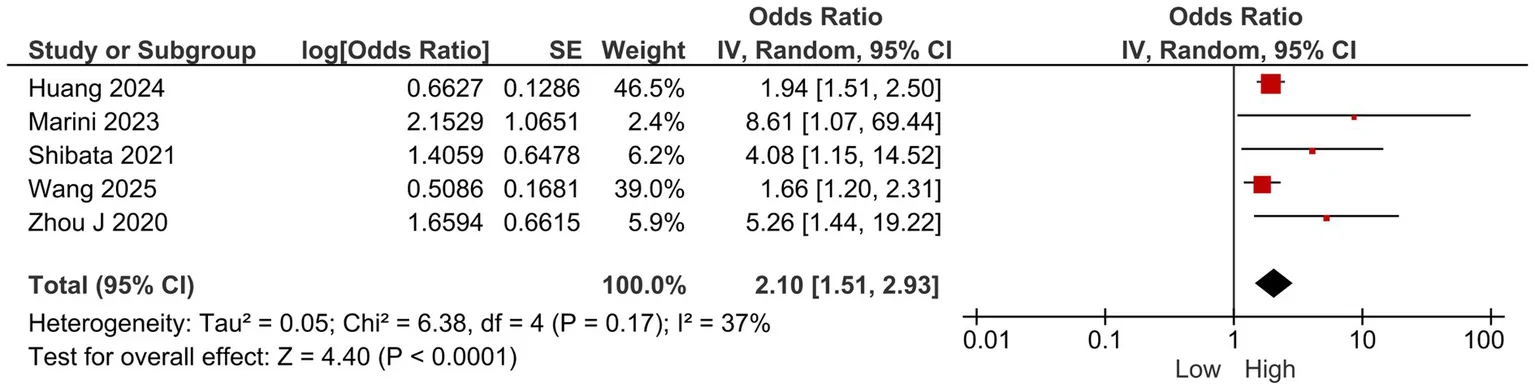
Prognostic value of GPR as a predictor for adverse TBI outcomes across five included studies.

### Subgroup analyses

1 Subgroup Analysis by Sample Size:

To investigate the source of heterogeneity in the mortality analysis (*I^2^* = 88%), studies were stratified by sample size (N > 500 vs. N < 500). Subgroup analysis suggested that sample size may have contributed substantially to the observed heterogeneity. The large registry-based study by Huang et al. (26) reported a more modest but statistically significant association between elevated GPR and mortality (OR 4.07, 95% CI 3.33–4.97). In contrast, the two smaller studies demonstrated substantially larger effect estimates, yielding a pooled OR of 25.26 (95% CI 10.87–58.72) without significant within-subgroup heterogeneity. The difference between subgroups was statistically significant (*p* < 0.0001), suggesting possible small-study effects. However, because only three studies contributed to this analysis, these findings should be interpreted cautiously.

- Test for Subgroup Differences: There was a statistically significant difference between the subgroups (*chi^2^* = 17.05, df = 1, *p* < 0.0001), with an I^2^ for subgroup differences of 94.1%. This suggests that sample size is a major contributor to the observed heterogeneity (Figure 5)

Subgroup Analysis by Ethnicity:

**Figure 5:**
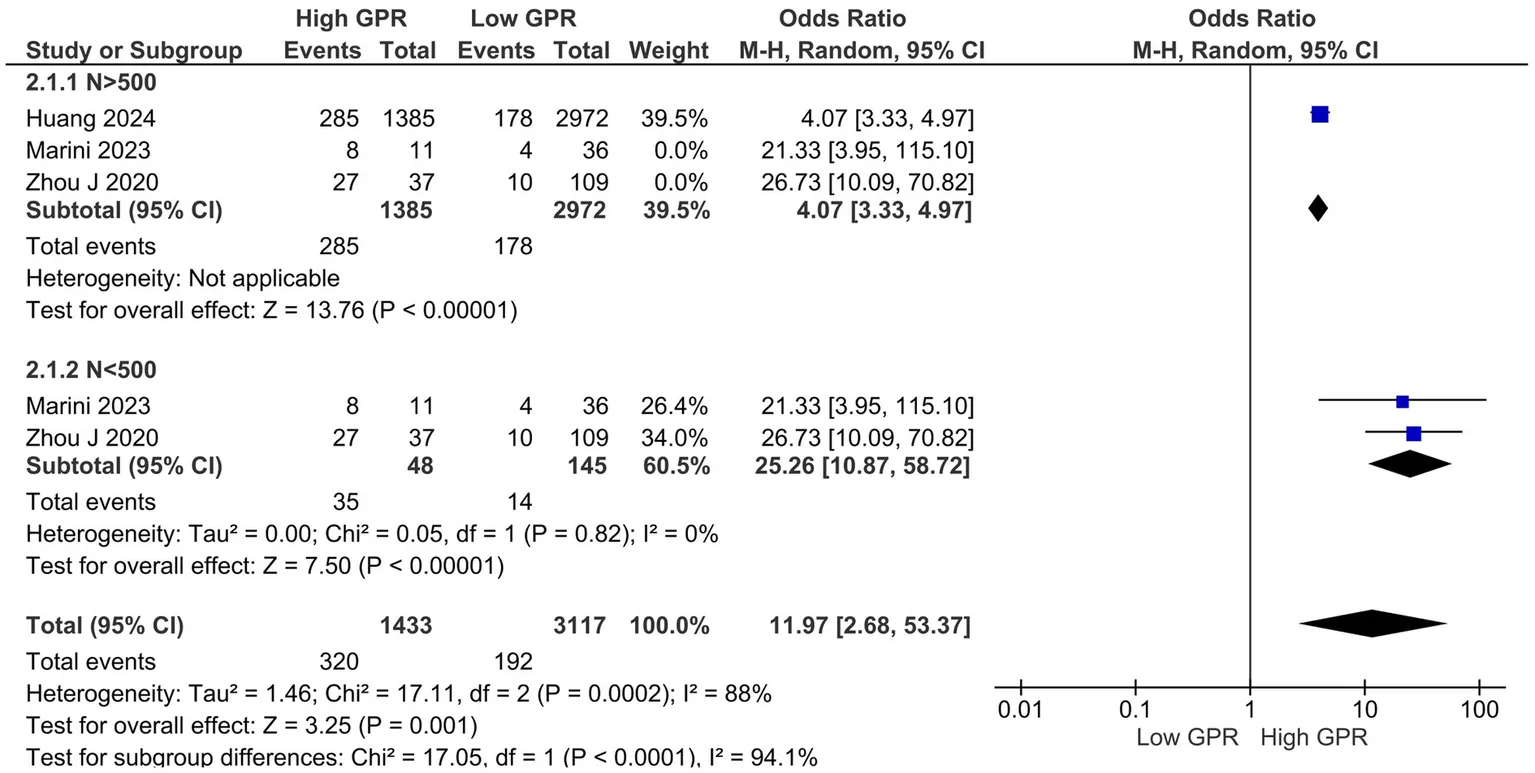
Subgroup analysis of mortality risk stratified by sample size (N > 500 vs. N < 500) showing significant subgroup differences (*p* < 0.0001).

Studies were stratified into Asian and Non-Asian populations.

- Asian Population: The pooled OR was 1.98 (95% CI: 1.48, 2.66; I2 = 32%).
- Non-Asian Population: The single study (25) reported a higher OR of 8.61 (95% CI: 1.07, 69.44).
- Test for Subgroup Differences: The difference between ethnicities was not statistically significant (chi2 = 1.86, df = 1, p = 0.17), although the interaction I2 was 46.3% (Figure 6)

**Figure 6:**
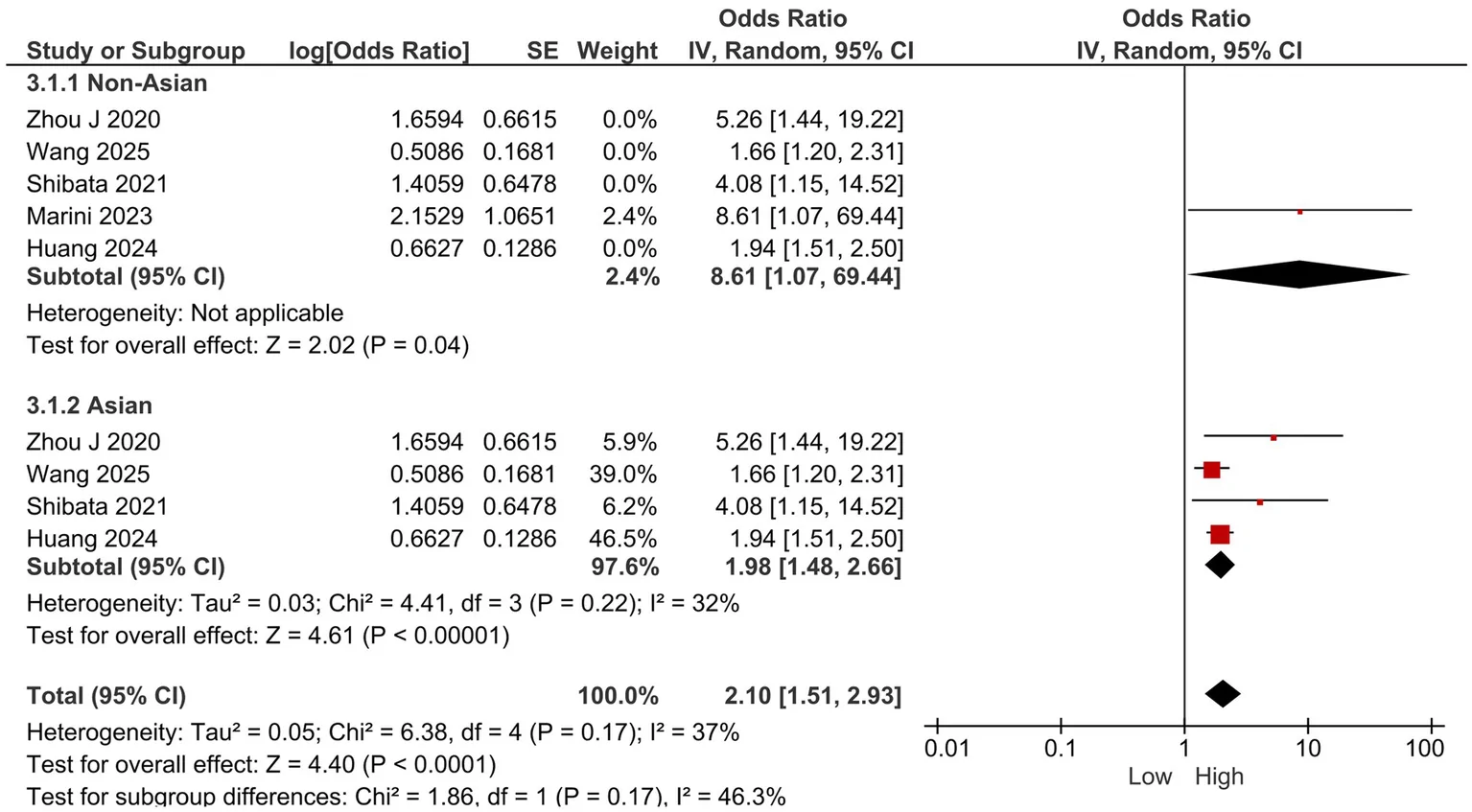
Subgroup analysis of mortality risk stratified by ethnicity (Asian vs. Non-Asian populations).

### Sensitivity analyses

1 Leave-One-Out Analysis (Exclusion of Large Sample Study):

Sensitivity analysis was performed by excluding the largest study (26) from the mortality analysis. The removal of this study resulted in a pooled OR of 25.26 (95% CI: 10.87, 58.72). Importantly, excluding Huang (26) completely resolved the heterogeneity (*I^2^* reduced from 88 to 0%, *p* = 0.82) (Figure 7)

2 Fixed-Effects Model Validation:

**Figure 7:**
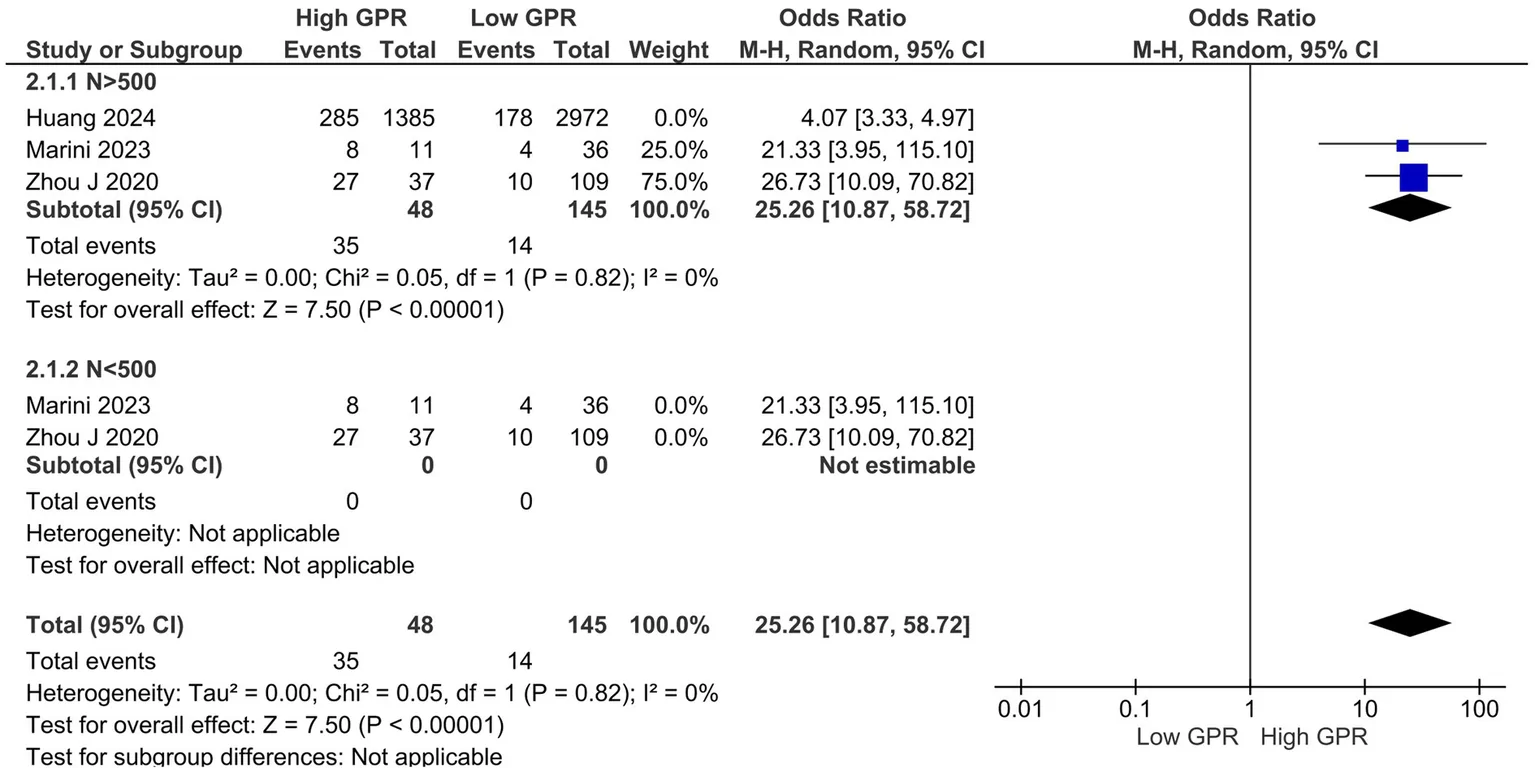
Sensitivity analysis (Leave-One-Out) excluding the largest study (26), resulting in the resolution of heterogeneity (I^2^ = 0%).

When re-analyzing the mortality data using a Fixed-Effects model instead of Random-Effects, the pooled OR decreased to 4.50 (95% CI: 3.71, 5.46)21. The heterogeneity remained significant (*I^2^* = 88%). The large discrepancy between the Random-Effects OR (11.97) and Fixed-Effects OR (4.50) further indicates the influence of small-study effects and high heterogeneity (Figure 8).

**Figure 8:**
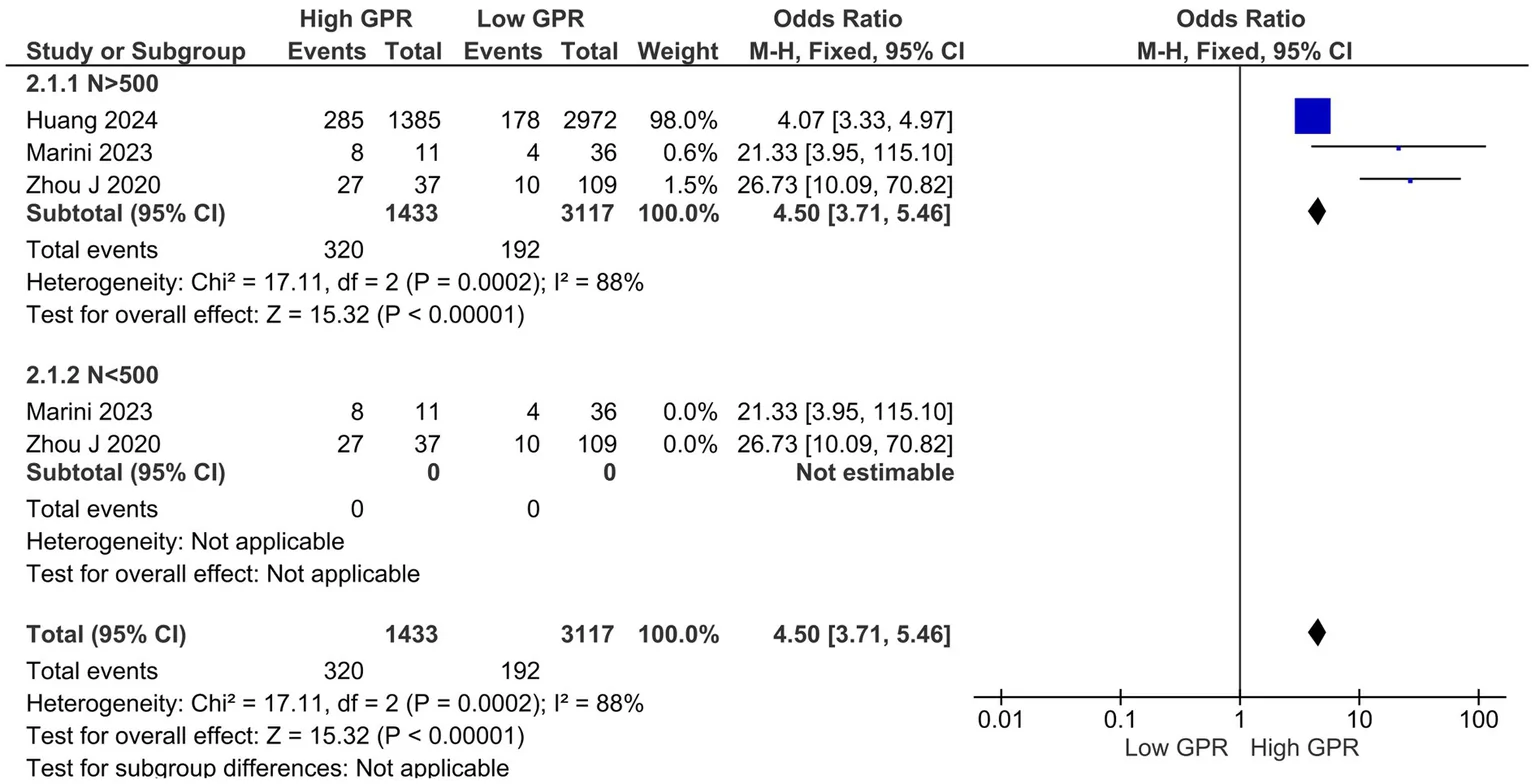
Sensitivity analysis using a fixed-effects model for the primary mortality outcome to validate model selection.

Publication Bias and Small-Study Effects

1 Assessment of Mortality Analysis (High vs. Low GPR)

Visual inspection of the funnel plot for the primary mortality outcome revealed marked asymmetry.

- Visual Pattern: The plot displays a distinct lack of the characteristic inverted “V” shape. The studies are widely scattered, with a notable gap in the bottom-left quadrant (representing small studies with low effect sizes).
- Small-Study Effect: There is a clear separation based on precision (Standard Error).

- The high-precision study (26) is located near the top left with a low Standard Error (*SE* < 0.2) and a moderate Odds Ratio.
- Conversely, the lower-precision studies (25, 27) are located in the bottom right, exhibiting high Standard Errors (*SE* ≈ 0.5–0.9) and substantially larger effect sizes (OR > 20).

- Interpretation: This distribution strongly suggests the presence of small-study effects, where smaller studies report significantly larger effect sizes than the larger study, contributing to the high heterogeneity (*I^2^* = 88%) observed in the Figure 9

2 Assessment of GPR Levels (Mean Difference)

**Figure 9:**
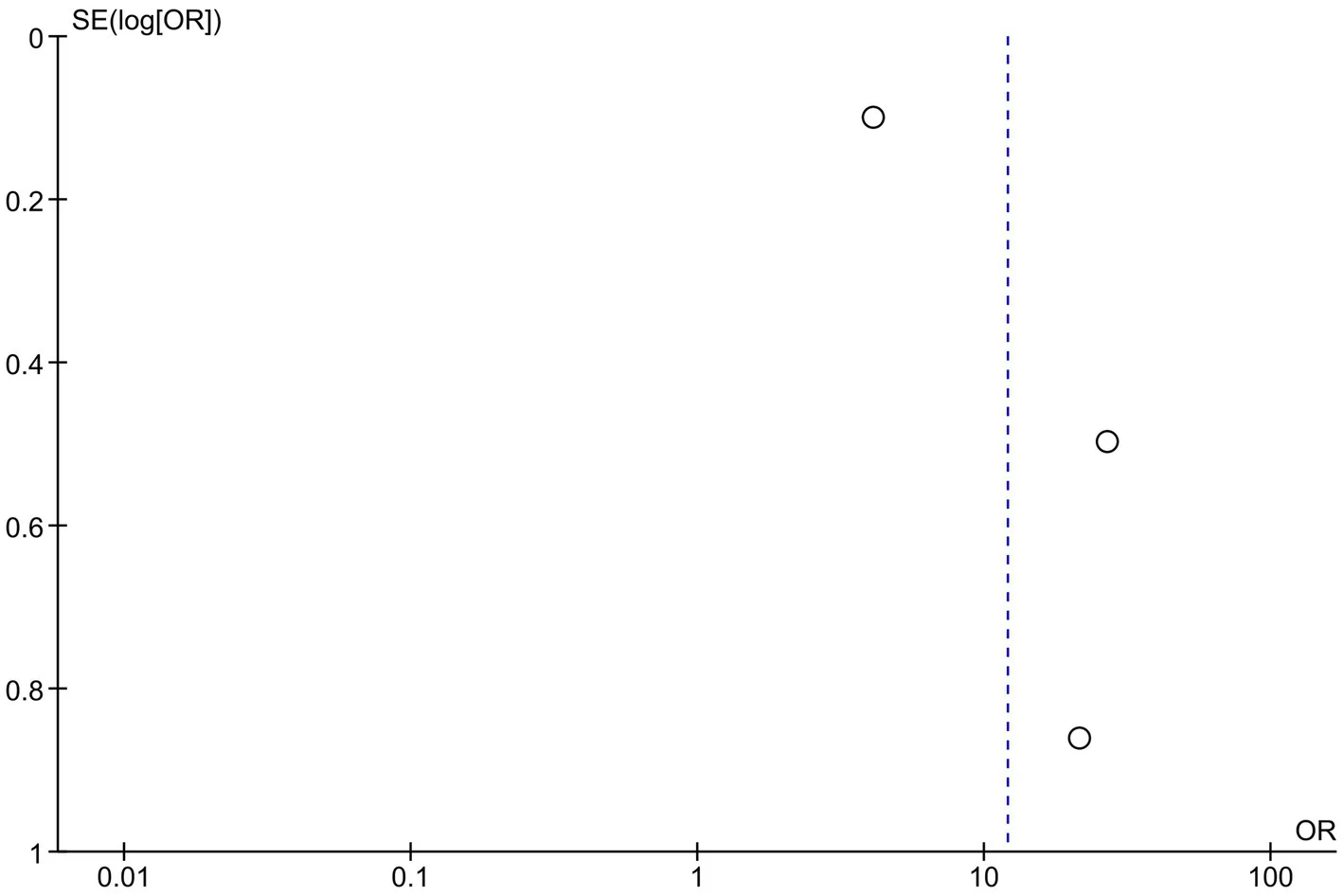
Visual assessment of publication bias for the primary outcome (GPR and Mortality). Asymmetry indicates the presence of small-study effects.

The funnel plot for the continuous outcome of GPR levels included four studies.

- Visual Pattern: The plot shows a vertical distribution of studies with Standard Errors ranging from approximately 1.5 to 3.5.
- Symmetry: While less asymmetrical than the mortality plot, the studies are not perfectly centered around the pooled Mean Difference. The study with the highest precision (lowest SE) pulls the effect size toward the center, while studies with higher SE [e.g., Zhou (27)] show larger deviations. The moderate heterogeneity (*I^2^* = 61%) noted in the main analysis is visually reflected here by the spread of the points beyond the central axis (Figure 10)

3 Subgroup Analysis Verification via Funnel Plots

**Figure 10:**
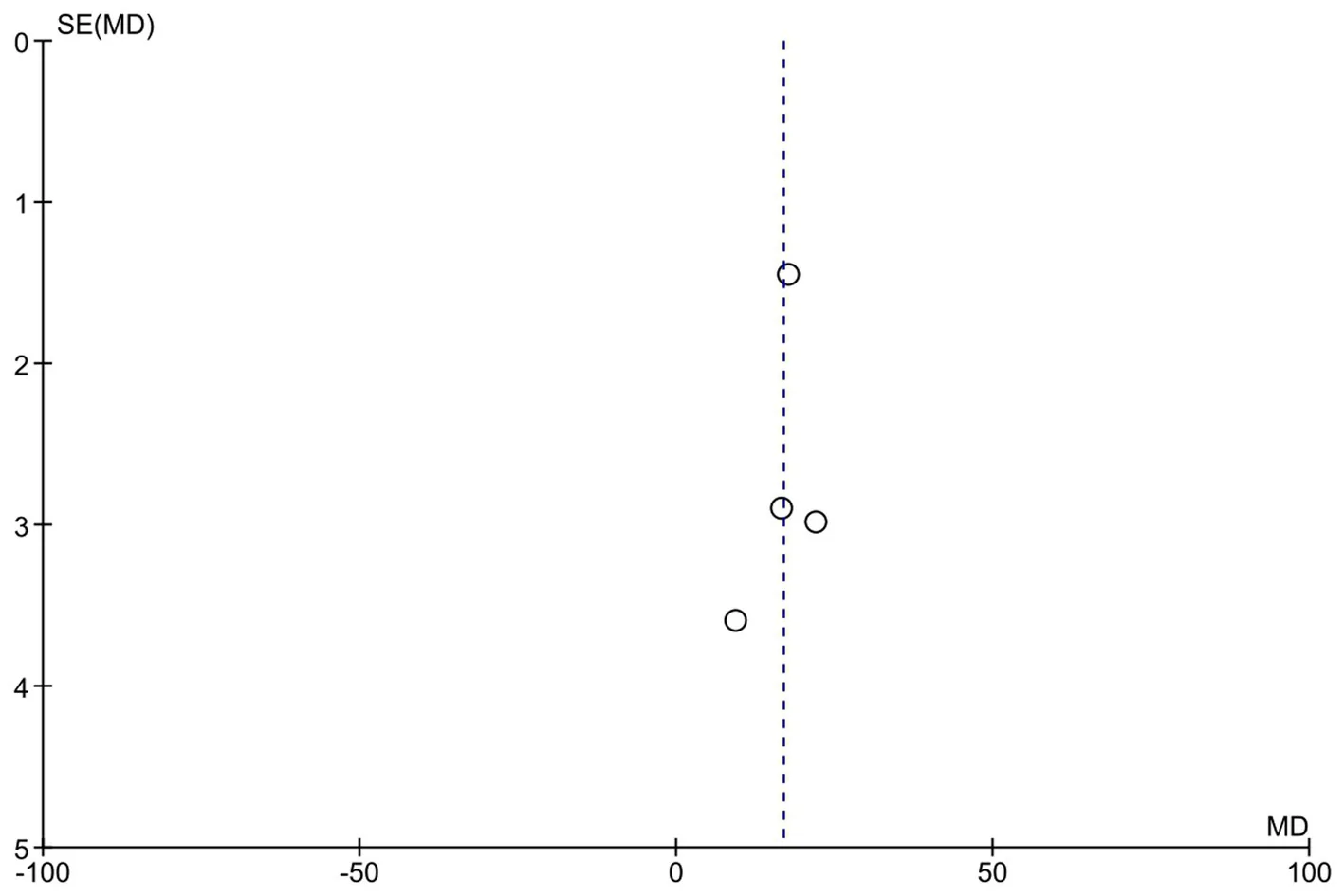
Visual assessment of publication bias for continuous outcomes (GPR levels).

We utilized funnel plots to visualize the impact of subgroup stratifications on the distribution of effect sizes.

- Subgroup by Sample Size:

- This plot provides visual confirmation of the source of heterogeneity. The subgroup stratification reveals two distinct clusters:

- (Circle): Represents the single large study (26), positioned at the top with high precision and a conservative effect estimate.
- (Diamonds): Represents the small studies, clustered at the bottom right with lower precision and inflated effect estimates.

- Numerical Implication: The distinct visual separation between the circle and diamonds on the x-axis confirms that sample size is a significant moderator of the effect size (Figure 11)

- Subgroup by Ethnicity:

- Asian Studies (Diamonds): These studies cluster vertically on the left side of the plot, indicating consistency in effect size among the Asian cohort.
- Non-Asian Study (Circle): The single non-Asian study appears as an outlier to the right, suggesting that geographic or ethnic differences may contribute to the variation in GPR prognostic value, though to a lesser extent than sample size (Figure 12)

4 Sensitivity Analysis Validation

- Fixed-Effects Model Check:

- The funnel plot overlaying the 95% pseudo-confidence limits (dashed lines) shows that the data points fall outside the triangular region.
- Interpretation: This confirms that a Fixed-Effects model is inappropriate for this data, as the observed variance between studies is far greater than what would be expected by sampling error alone. This validates the decision to report the Random-Effects model in the primary analysis (Figure 13)

- Leave-One-Out Sensitivity:

- After removing the largest study (26), the remaining two points align vertically with high Standard Errors (*SE* > 0.5). While this restores symmetry (as both have high ORs), the analysis relies solely on low-precision studies, reinforcing the importance of including the large-sample study for a balanced weight analysis (Figure 14)

**Figure 11:**
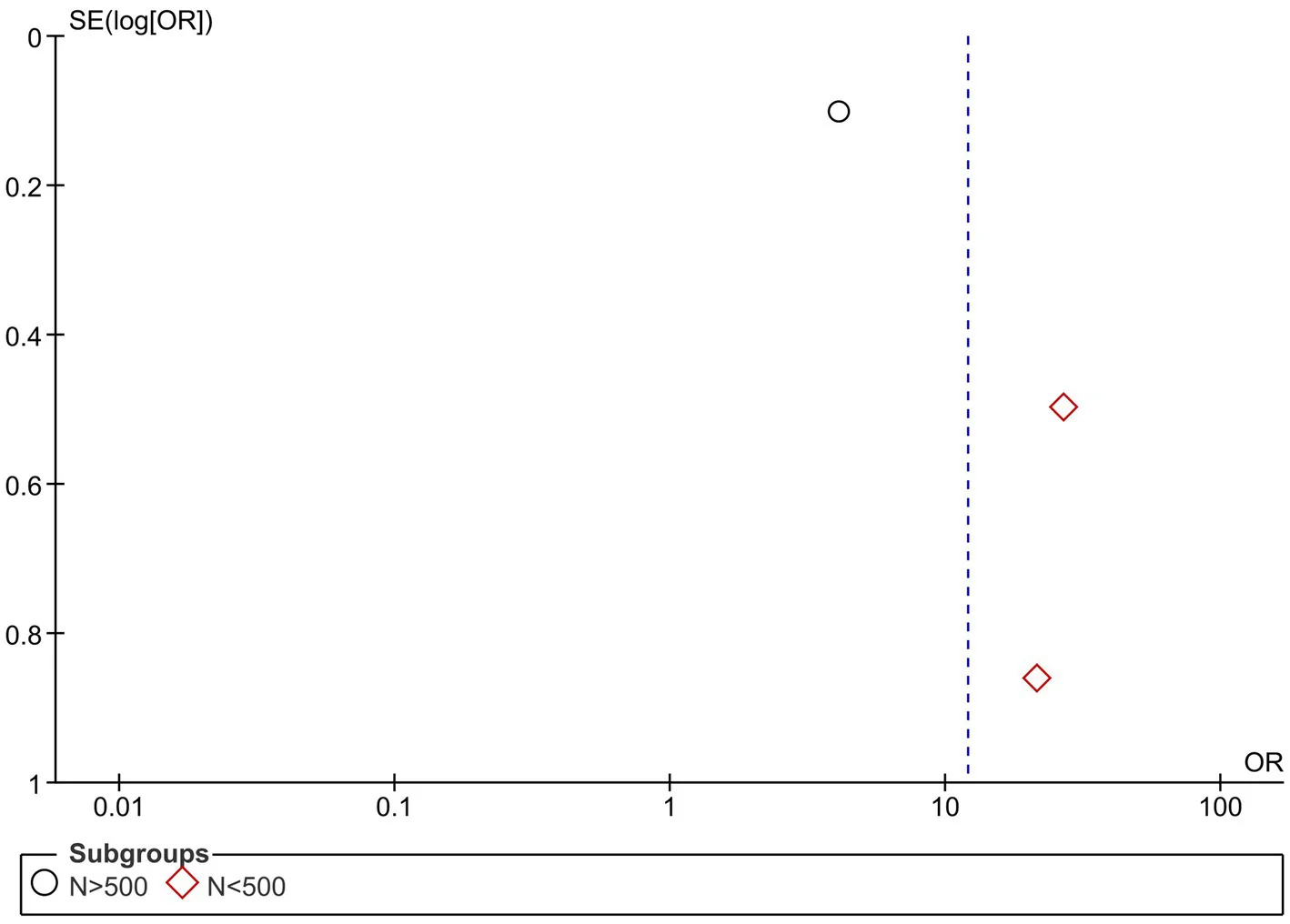
Funnel plot by sample size subgroup, showing distinct clustering of small studies with lower precision and higher effect sizes compared to the large study.

**Figure 12:**
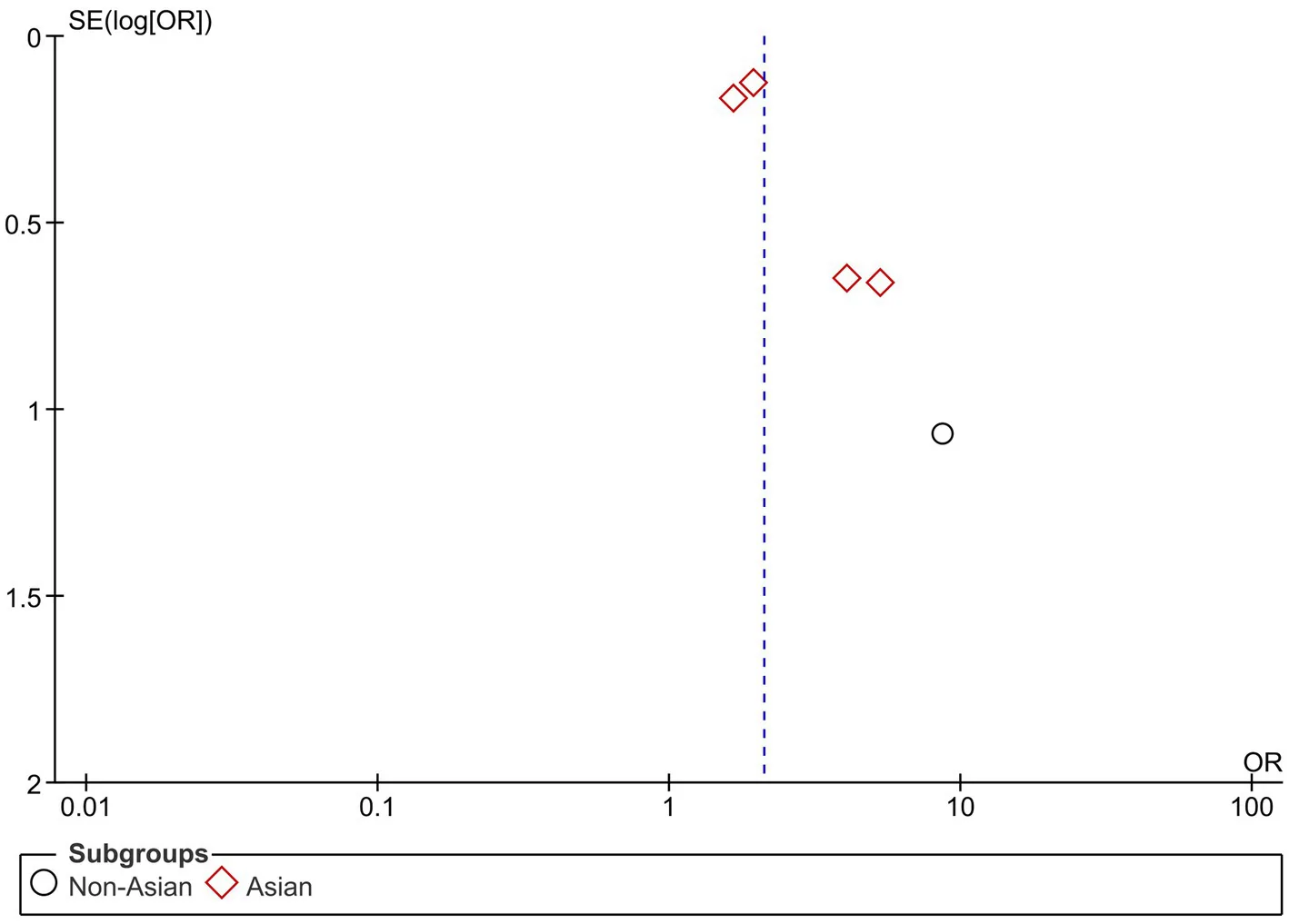
Funnel plot by ethnicity subgroup, visualizing the distribution of Asian versus Non-Asian studies.

**Figure 13:**
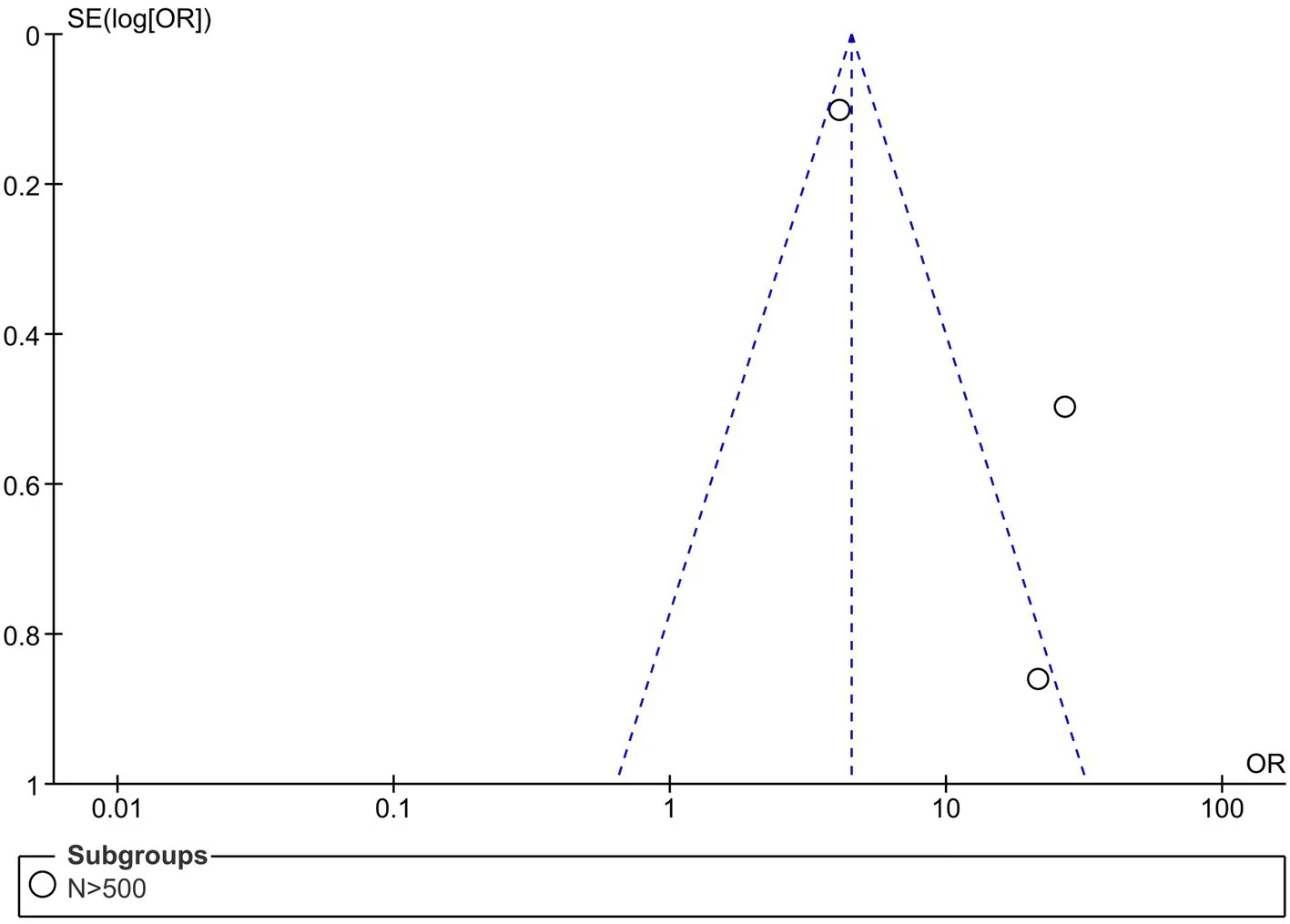
Funnel plot validating the random-effects model; data points falling outside the pseudo-confidence limits of the fixed-effects model confirm high heterogeneity.

**Figure 14:**
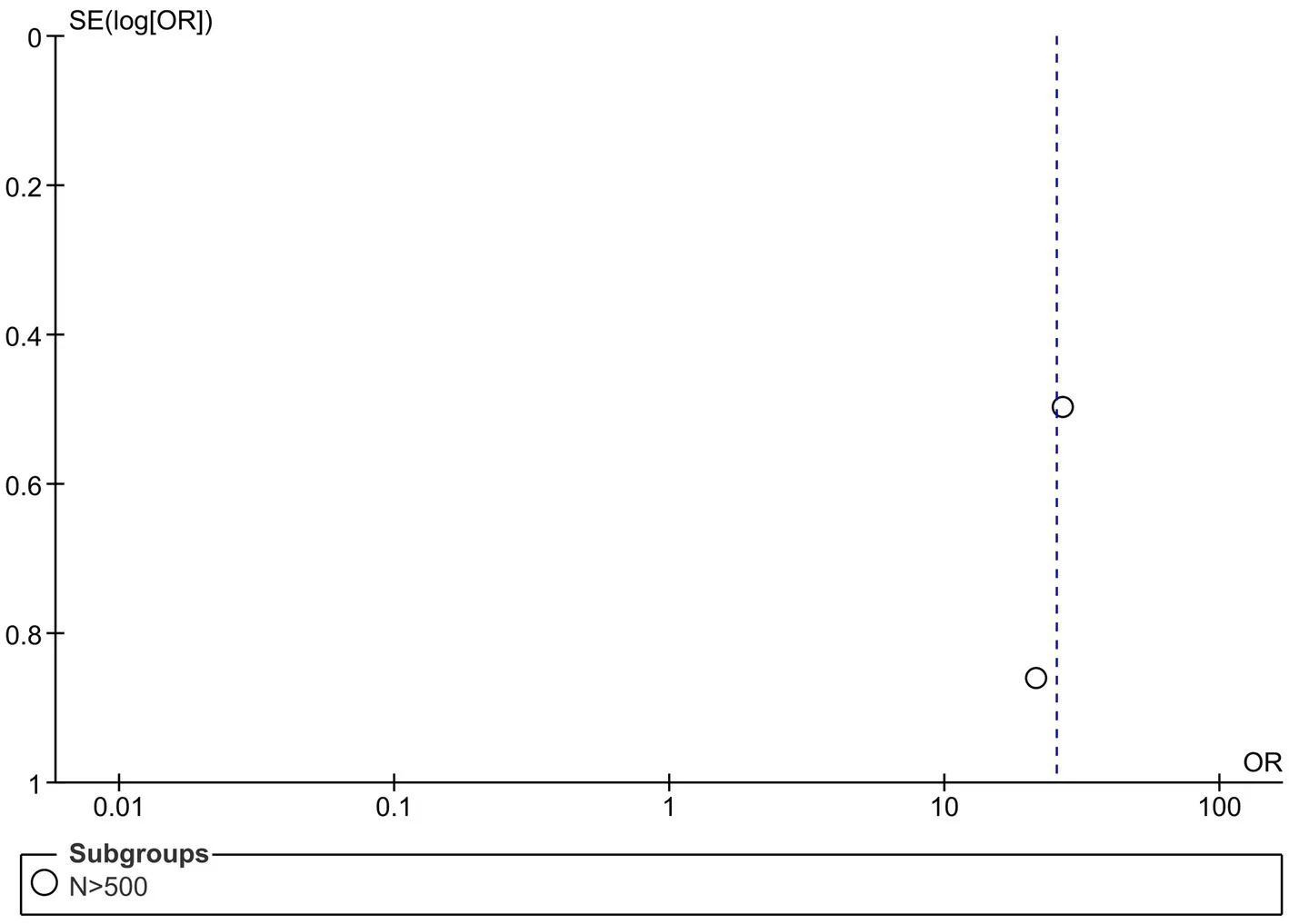
Funnel plot for the leave-one-out sensitivity analysis [excluding Huang (26)], showing the distribution of the remaining lower-precision studies.

### Conclusion of publication bias assessment

Due to the limited number of studies (*k* < 10), statistical tests for funnel plot asymmetry (such as Egger’s regression) may lack power; however, the visual inspection strongly indicates that small-study effects are driving the high effect sizes observed in the mortality analysis. Results should be interpreted with caution regarding the magnitude of the Odds Ratio in smaller cohorts.

## Discussion

In this systematic review and meta-analysis of adult traumatic brain injury (TBI), we found that an elevated admission glucose-to-potassium ratio (GPR), or stress index (SI), is consistently associated with worse clinical outcomes. Patients with high GPR had markedly increased odds of mortality compared with those with low GPR, and individuals with poor functional outcomes exhibited significantly higher mean GPR values. Furthermore, GPR remained an independent predictor of adverse outcomes across multiple studies, supporting its role as a robust indicator of early systemic stress and injury severity.

These findings align closely with prior cohort studies that first proposed GPR or SI as a prognostic biomarker in TBI. Shibata et al. reported that a GPR ≥ 50 on arrival was associated with poor outcome (death or vegetative state), even after adjustment for age and GCS (24). Marini et al. (25) observed a similar threshold effect in patients with moderate-to-severe TBI and suggested that GPR may complement CT-based prognostic models. Huang et al. (26) further demonstrated that SI independently predicted mortality in isolated moderate-to-severe TBI (26). Additional studies in patients undergoing emergency craniotomy and in mild-to-moderate TBI populations have also shown that elevated GPR is associated with increased mortality and unfavorable neurological outcomes (27, 28). The present meta-analysis synthesizes these findings and provides a quantitative estimate of the overall effect size across heterogeneous TBI populations.

The biological plausibility of GPR as a prognostic marker is well supported. Severe TBI triggers a complex neuroendocrine response involving activation of the sympathetic nervous system and the hypothalamic–pituitary–adrenal (HPA) axis, leading to catecholamine and cortisol surges (2, 4). These responses promote hepatic gluconeogenesis, glycogenolysis, and insulin resistance, resulting in stress hyperglycemia, which has been consistently associated with increased mortality and poor neurological outcomes (6–8). Concurrently, β2-adrenergic stimulation and insulin-mediated cellular uptake of potassium lead to intracellular shifts and hypokalemia (11). Electrolyte disturbances, particularly dyskalemia, have been linked to arrhythmias, hemodynamic instability, and worse outcomes in TBI (12–15).

Importantly, GPR does not merely reflect two independent abnormalities but rather integrates these processes into a single composite biomarker of systemic stress. It is plausible that elevated GPR identifies a specific phenotype of TBI patients characterized by exaggerated neuroendocrine activation or hypothalamic–pituitary dysfunction. Such patients may experience more severe secondary brain injury through mechanisms including metabolic crisis, oxidative stress, and microvascular dysfunction (2, 4, 14). Compared with glucose alone, which reflects only one dimension of the stress response, GPR incorporates electrolyte dynamics and may therefore provide improved prognostic discrimination. This is consistent with prior studies demonstrating that composite biomarkers often outperform individual variables in predicting clinical outcomes (18, 24, 25).

Evidence from related acute conditions further supports the prognostic utility of GPR. In intracerebral hemorrhage, elevated GPR has been associated with worse functional outcomes and improved predictive performance when added to conventional models (18). Large trauma registry studies have shown that SI independently predicts in-hospital mortality in unselected trauma patients (20). Similarly, studies in sepsis, cardiovascular emergencies, and critically ill populations have demonstrated that elevated GPR or SI is associated with increased mortality and complications (19, 21–23). These findings suggest that GPR captures a generalized physiological stress response that is applicable across diverse acute care settings. Other readily available laboratory biomarkers have also been investigated for prognostication in traumatic brain injury. Among these, the De Ritis Ratio (AST/ALT ratio) has emerged as a promising predictor of mortality and unfavorable neurological outcomes. Elevated De Ritis Ratio values are thought to reflect systemic metabolic stress, hepatic dysfunction, mitochondrial injury, and secondary inflammatory responses following severe trauma. Similar to GPR, the De Ritis Ratio can be rapidly calculated from routine laboratory tests and has demonstrated independent prognostic value in several TBI cohorts. However, direct comparisons between GPR and the De Ritis Ratio remain limited. Future studies should evaluate whether combining these biomarkers improves predictive accuracy beyond established clinical and radiological prognostic models (32). Nevertheless, GPR should not be regarded as a TBI-specific biomarker. Because both glucose and potassium levels are influenced by systemic physiological stress, elevated GPR may reflect the severity of critical illness rather than injury-specific pathophysiology. Factors such as polytrauma, hemorrhagic shock, infection, endocrine disorders, diabetes mellitus, renal dysfunction, and prehospital interventions may influence GPR independently of brain injury severity. Consequently, residual confounding remains an important consideration, and GPR should be interpreted alongside established clinical, radiological, and prognostic models rather than as a standalone predictor.

Despite these consistent associations, substantial heterogeneity was observed in the primary mortality analysis (I^2^ = 88%). Subgroup analysis indicated that sample size was a major contributor, with smaller studies reporting markedly larger effect sizes compared to larger registry-based cohorts. This pattern is consistent with small-study effects and potential publication bias, where smaller single-center studies may overestimate associations (20, 23, 26). In addition, variability in GPR cut-off values likely contributed significantly to heterogeneity. Reported thresholds ranged widely—from approximately 2.28 in mmol/L-based measurements to ≥50 in mg/dL ÷ mEq/L systems—reflecting differences in laboratory units and study design (18, 19, 24, 25, 27, 30). Differences in TBI severity (e.g., GCS strata), inclusion criteria, and outcome definitions may also influence effect size; however, limited reporting and the small number of studies precluded formal subgroup meta-analysis based on these factors.

From a clinical perspective, the lack of standardized GPR cut-off values remains a major limitation. While several studies suggest that thresholds around 45–50 may best discriminate high-risk patients in conventional units (24, 25), sensitivity and specificity data were inconsistently reported, limiting direct comparison. This variability highlights the need for standardized threshold validation in large, prospective, multicenter cohorts before GPR can be reliably incorporated into clinical decision-making.

Another important limitation is the observational nature of the included studies, most of which were retrospective. Such designs are inherently subject to selection bias, measurement variability, and residual confounding. Key variables that may influence GPR—such as pre-hospital fluid resuscitation, timing from injury to blood sampling, insulin administration, and comorbid conditions (e.g., diabetes or renal dysfunction)—were not consistently controlled across studies (6–8, 10, 33). These factors may independently affect glucose and potassium levels and thereby influence the calculated ratio. Additionally, although some studies restricted analyses to isolated TBI or adjusted for extracranial injuries, polytrauma remains common in severe TBI and may confound observed associations (16, 17).

Geographical distribution of included studies represents another limitation. Most studies were conducted in Asian populations, with limited representation from other regions. Differences in baseline metabolic profiles, healthcare systems, and trauma management protocols may influence both GPR values and outcomes, potentially limiting the generalizability of these findings to other populations.

An emerging area of interest is the dynamic trajectory of GPR over time. All included studies evaluated GPR at admission; however, evolving evidence suggests that persistent metabolic derangements may have greater prognostic significance than transient abnormalities. For example, sustained hyperglycemia has been associated with worse outcomes compared to transient elevations in TBI populations (10). It is plausible that persistent elevation of GPR reflects ongoing neuroendocrine dysregulation and secondary injury processes, whereas normalization may indicate physiological recovery. Future studies should investigate serial GPR measurements and temporal patterns to enhance risk stratification.

Importantly, GPR should be interpreted as a prognostic biomarker rather than a direct therapeutic target. Although elevated GPR is associated with worse outcomes, it likely reflects underlying systemic stress rather than a causal mediator of injury. Interventional studies targeting tight glycemic control have shown inconsistent or harmful results in TBI and critical illness, emphasizing the complexity of metabolic management (10, 33). Similarly, aggressive correction of potassium disturbances carries risks and must be approached cautiously (11, 16, 17). Therefore, GPR is best viewed as a tool for early risk stratification rather than a parameter requiring direct intervention.

Several limitations should be acknowledged. First, all included studies were observational in nature, with most employing retrospective designs, making them susceptible to selection bias, residual confounding, and measurement bias. Second, the overall evidence base remains limited by the relatively small number of available studies and the predominance of single-center cohorts. Third, substantial heterogeneity was observed in the primary mortality analysis, likely reflecting differences in study populations, injury severity, laboratory measurement units, outcome definitions, and GPR cutoff values. Fourth, most studies originated from Asian populations, which may limit the generalizability of findings to other healthcare settings and ethnic groups. Finally, evidence of small-study effects and potential publication bias was observed, suggesting that the magnitude of the pooled effect may be overestimated. This review was not prospectively registered in PROSPERO, which may limit methodological transparency and increase the potential risk of selective reporting. No professional librarian was involved in designing or validating the search strategy, which may have affected the comprehensiveness of literature retrieval despite searching multiple databases and performing manual reference screening.

A major practical advantage of GPR is that it can be calculated immediately from routine admission laboratory tests without additional cost, specialized equipment, or delays in obtaining results, making it particularly attractive for emergency and resource-limited settings. Overall, GPR/SI represents a simple, low-cost, and readily available biomarker derived from routine laboratory tests. Its ability to integrate metabolic and electrolyte disturbances into a single index makes it an attractive adjunct to established clinical and radiological predictors in TBI. However, given the observed heterogeneity, small-study effects, limited number of included studies, and lack of standardized thresholds, further large-scale, multicenter prospective studies are required. Future research should focus on standardizing measurement units, validating optimal cut-offs, accounting for confounding variables, and exploring dynamic changes over time to fully establish the clinical utility of GPR in TBI.

## Conclusion

This systematic review and meta-analysis suggest that elevated admission glucose-to-potassium ratio (GPR) is associated with increased mortality and unfavorable functional outcomes in adult patients with traumatic brain injury. As a composite marker integrating stress hyperglycemia and hypokalemia, GPR may reflect the intensity of the systemic neuroendocrine response to injury. However, these findings should be interpreted cautiously because the available evidence is derived predominantly from retrospective observational studies with heterogeneous methodologies, variable cutoff values, and potential small-study effects. Consequently, GPR should currently be considered a promising prognostic biomarker rather than a definitive clinical decision-making tool. Large prospective multicenter studies are needed to validate standardized thresholds and determine whether GPR provides incremental prognostic value beyond established clinical and radiological models.

## Data Availability

All data analyzed were extracted from published studies and are reported in the manuscript and/or supplementary files. Additional extracted data sheets and analytic outputs are available upon reasonable request.
